# Self-reported diabetes during pregnancy in the South African National Health and Nutrition Examination Survey: extent and social determinants

**DOI:** 10.1186/s12884-016-1218-z

**Published:** 2017-01-10

**Authors:** Lumbwe Chola, Chipo Mutyambizi, Ronel Sewpaul, Whadi-ah Parker, Zandile Mchiza, Demetre Labadarios, Charles Hongoro

**Affiliations:** 1 Population Health, Health Systems and Innovation, Human Sciences Research Council, HSRC Building, 134 Pretorius Street, Pretoria, 0002 South Africa; 2 School of Public Health, Faculty of Health Sciences, University of the Witwatersrand, Johannesburg, South Africa

**Keywords:** Diabetes, Pregnancy, South Africa, Social determinants

## Abstract

**Background:**

Diabetes is a serious and growing public health concern in South Africa, but its prevalence and distribution in pregnant women is not well known. Women diagnosed with diabetes during pregnancy have a substantially greater risk of adverse health outcomes for both mother and child. This study aims to determine the prevalence and social determinants of diabetes during pregnancy in South Africa.

**Methods:**

Data used in this study were from the 2012 South African National Nutrition and Health Examination Survey; a nationally representative cross-sectional household survey. The analysis was restricted to girls and women between the ages of 15 to 49 years who self-reported ever being pregnant (*n* = 4261) Logistic regression models were constructed to analyse the relationship between diabetes during pregnancy and several indicators including race, family history of diabetes, household income, area of residence and obesity.

**Results:**

The prevalence of diabetes during pregnancy in South Africa was 3% (144 women) of all women who reported ever being pregnant. The majority of the women who had ever had diabetes were African (70%), 51% were unemployed and 76% lived in rural areas. Factors strongly associated with diabetes during pregnancy were age (1.04 [Odds Ratio], 0.01 [Standard Error]), family history of diabetes (3.04; 0.8) and race (1.91; 0.53).

**Conclusion:**

The analysis will contribute to an understanding of the prevalence of diabetes during pregnancy and its social determinants. This will help in the development of effective interventions targeted at improving maternal and child health for mothers at high risk.

## Background

The global burden of diabetes mellitus, a chronic metabolic disorder caused by defects in insulin production, has continued to rise with rapid increases observed in low and middle income countries [1, 2]. Globally, the population with diabetes is projected to rise to a high of 592 million in 2035, from 285 million in 2010 [3, 4]. In South Africa, adult diabetes prevalence was estimated to be approximately 9% in 2009 [5]. With these trends, there has also been an increase in the prevalence of diabetes during pregnancy, which is a source of major concern for countries worldwide [6, 7].

Diabetes in pregnancy can either be pre-existing type 1 or type 2 diabetes, or gestational diabetes mellitus (GDM), which is defined as glucose intolerance of variable severity that is first recognised during pregnancy [8]. It is estimated that type 2 diabetes affects about 92 million women of reproductive age worldwide, and up to 14% of pregnant women will have GDM [9–11]. In pregnant women and their children, diabetes is associated with increased risk of hypertension, miscarriages, stillbirths, prematurity and macrosomia among others [8, 12–14].

Not much is known about the prevalence and distribution of diabetes in pregnant South African women. An audit of pregnancy outcomes conducted at Chris Hani Baragwanath Hospital in Soweto between 1992 and 2002 suggests that approximately 2% of screened pregnant women had diabetes [15]. A programme implemented at the hospital to manage diabetes in pregnancy was estimated to reduce perinatal mortality by 25%. Two studies conducted in sub-populations in Johannesburg and Limpopo found a diabetes prevalence of 1.8% and 8.8% in pregnant women [16, 17].

This study uses self-reported data from a nationally representative survey to estimate the extent of diabetes during pregnancy in South Africa women. The study also assesses the social determinants of diabetes in pregnancy. The analysis adds to the limited literature and can be used to inform policy and priority setting to improve pregnancy outcomes.

## Methods

The South African National Health and Nutrition Examination Survey (SANHANES), was a cross-sectional survey undertaken in 2012, to measure the nutrition and health status of the South African population. The survey employed a multi-stage, stratified cluster sampling approach. The Master Sample was created by selecting a total of 1000 census enumeration areas (EAs) mapped using aerial photography in 2007. The selection of EAs was stratified by province and locality type. A total of 500 EAs representative of the socio-demographic profile of South Africa were selected from the Master Sample. From each EA, random samples of 20 visiting points (VPs) were then selected, yielding an overall sample of 10 000 households. The final sample consisted of 8,166 households, with 27,580 eligible individuals, of which 92.6% (25 532) participated in the survey. Details of the survey are given elsewhere [18, 19].

In this paper, the analysis was restricted to girls and women between the ages of 15 to 49 years who reported having ever been pregnant. A total of 4261 participants within the reproductive years of 15–49 years indicated that they had been pregnant before, of which 144 (3%) reported having had diabetes during pregnancy. In the analysis the dependant variable was self-reported diabetes during pregnancy, which was coded into a binary variable with 0 indicating having ever been pregnant and not diabetic during pregnancy and 1 indicating ever pregnant and diabetic during pregnancy.

We assessed the social determinants of diabetes in pregnancy, with variable selection guided by the conceptual framework for the social determinants of health suggested by the WHO Commission on Social Determinants of Health (CSDH) [20]. This model explains the structural and intermediary factors that influence health and wellbeing. Structural determinants include factors related to socioeconomic status, such as education and income, while intermediary determinants of health include material circumstances of living and food availability, biological and psychosocial factors.

The structural determinants included in the analysis were: age, race, education, employment status, residence and household income. Age was measured in years and included as a continuous variable. The SANHANES collected data on the four main ethnic groups in South Africa – Africans, Coloureds, Whites and Indians. Very few non-African women reported having diabetes during pregnancy, as a result race was recoded into a binary variable as 0-African and 1-non-African. Education was categorised as 0- no education, 1- primary (1–7 years), 3- secondary (8–12 years) and 4- tertiary (13+ years). Employment had three categories: unemployed, informal and formal employment. Residence was categorised into 0-rural and 1-urban. A continuous annual household income variable was derived from the categorical data collected in the SANHANES for each adult member of the household, by taking the mid-point estimate of each income category. We then estimated the total income and divided this by the number of adults to get the average income in each household. Average household income was re-categorised into quantiles denoting low, medium and high.

The intermediary determinants included in the analysis were family history of diabetes and self-reported health. Family history of diabetes was self-reported. Respondents in the SANHANES survey were also asked to rate their general health status. This variable was included in this analysis as a categorical variable with 0 denoting bad, 1- moderate and 2- good.

Statistical analyses were performed in STATA software version 13 (Stata Corp. Inc. TX, USA). Clustering and survey design effects were accounted for using Stata’s stratified multi-stage design command. In reporting the descriptive statistics, means and standard deviations are presented for continuous variables and proportions for categorical variables.. Unadjusted and adjusted odds ratios were calculated to estimate the strength of association, with significance levels set at *p* < 0.05.A logistic regression was fitted using a stepwise method guided by the CSDH model. Step 1 was a univariate or unadjusted analysis; step 2 included structural determinants; step 3 included intermediate determinants; and step 4 included all variables that were significant in steps 2 and 3.

Ethical approval for the SANHANES was obtained from the Research Ethics Committee of HSRC (REF: EC6/16/11/11). Written consent was obtained from participants. Authorised consent, signed by parents or guardians was obtained for persons under 18 years.

## Results

A total of 144 women (3% of women 15–49 years who had ever been pregnant) reported having diabetes during pregnancy (Table 1). The mean age (standard deviation – SD) was 33 (9.1) years. Of the 144 women who reported diabetes in pregnancy, 70% were African.. Over 80% of those reporting diabetes during pregnancy had more than 7 years of schooling (secondary or tertiary education). 51% of women who had diabetes during pregnancy were unemployed and 76% lived in rural areas. More than half of the women who had diabetes during pregnancy had a family history of the disease.

**Table 1:** Characteristics of women who had diabetes during pregnancy

Variables	Diabetes in pregnancy	
	Number (*n* = 144)	Percent (100%)
Race		
African	82	70%
non-African	62	30%
Education		
None	3	1%
Primary	18	15%
Secondary	90	67%
Tertiary	14	17%
Employment		
Unemployed	66	51%
Informal	23	15%
Formal	53	34%
Residence		
Rural	108	76%
Urban	36	24%
Household income		
Low	54	40%
Medium	49	34%
High	41	26%
Family history of diabetes		
No	68	49%
Yes	70	51%
Self-rated health		
Bad	11	9%
Moderate	27	20%
Good	105	71%

Table 2 shows the factors associated with reported diabetes in pregnancy. In the unadjusted logistic regression model (step 1), age (1.05 [Odds Ratio]; 0.01 [Standard Error]), race (2.06; 0.5), family history (3.57; 0.85) and primary (4.18; 2.89), secondary (3.53; 2.15) and tertiary (4.48; 3.08) education were positively related to diabetes in pregnancy, with statistically significant associations. Those living in urban areas (0.62; 0.15) had lower likelihood of reporting diabetes, with statistically significant associations. After adjusting for structural factors in step 2, age (1.05; 0.01), race (1.91; 0.59) and secondary education (3.76; 2.37) remained statistically significant. Strong associations were also maintained for persons in the high household income category (0.51; 0.18), who were less likely to report diabetes. In step 3 (adjusting for intermediary factors), those reporting a family history of diabetes (3.6; 0.9) were strongly associated with having diabetes in pregnancy. In the final model (adjusted for structural and intermediary factors), age (1.04; 0.01), race (1.91; 0.53), and family history (3.04; 0.8) were significantly associated with diabetes in pregnancy. Non-Africans and those with family history had a higher likelihood of reporting diabetes.

**Table 2:** Determinants of diabetes in pregnancy

Characteristic	Step 1		Step 2		Step 3		Step 4	
	OR	SE	OR	SE	OR	SE	OR	SE
Age	1.05**	0.01	1.05**	0.01			1.04**	0.01
Race								
African	1		1				1	
non-African	2.06**	0.5	1.91*	0.59			1.91*	0.53
Education								
None	1		1				1	
Primary	4.18*	2.89	3.41	2.32			2.54	1.81
Secondary	3.53*	2.15	3.76*	2.37			2.68	1.72
Tertiary	4.48*	3.08	4.14	3.11			3.09	2.29
Employment								
Unemployed	1		1					
Informal	1.32	0.46	0.82	0.36				
Formal	1.08	0.27	0.94	0.28				
Residence								
Rural	1		1					
Urban	0.62*	0.15	0.69	0.21				
Household income								
Low	1		1				1	
Medium	0.78	0.22	0.78	0.23			0.77	0.24
High	0.74	0.21	0.51*	0.18			0.58	0.2
Family history								
No	1				1		1	
Yes	3.57**	0.85			3.6**	0.9	3.04**	0.8
Self-rated health								
Bad	1				1			
Moderate	0.63	0.31			0.67	0.33		
Good	0.45	0.2			0.46	0.2		

*P < 0.05; **P < 0.01; *OR* odds ratio, *SE* standard error

## Discussion

This nationally representative study recorded the reports of diabetes during pregnancy in South African women. Out of the 4,261 women between the years 15 to 49 who reported having been pregnant, 3% indicated that they had diabetes during their pregnancy. This estimate is within the global estimates for diabetes during pregnancy, and the range of 2% to 8% reported in South African populations [15–17]. There is not much in the literature on diabetes among pregnant South African women and more investigations are needed in this area. The findings in this study provide a snapshot of the problem and the likely social determinants of diabetes in pregnancy among South Africans. To our knowledge, this is the first study in South Africa, which indicates these associations at a population level. The findings will be important to healthcare planning and decision making and give impetus for increased attention to the prevention and management of diabetes in pregnancy.

Strong associations were observed for race and family history. Non-Africans were more likely to report diabetes in pregnancy than Africans. This has been shown to be the case in studies of type-2 diabetes in the general population, which report higher prevalence of type-2 diabetes in South Africans of non-African origin [21–23]. Our estimates also indicate higher likelihood of diabetes during pregnancy in rural women and those residing in lower income households. Future research should consider investigating these and other special populations that seem to be prone to diabetes.

A major strength of this study is the use of nationally representative data, which allowed for the examination of the social determinants of self-reported diabetes in pregnancy. The study is however limited in that causal inferences cannot be drawn due to the cross-sectional nature of the SANHANES. The prevalence of diabetes during pregnancy was self-reported and not measured, thus it was not possible to independently verify health status. We were also unable to classify the type of diabetes that was reported. It is also important to note that respondents were required to report whether they had diabetes during pregnancy regardless of the period in which this occurred, as a result, the study focused on the history of diabetes in pregnant women and not the current prevalence. Because of this, we did not include other risk factors for diabetes such as obesity and physical exercise, which would have been informative if measured together with the diabetes.

Diabetes in pregnancy presents major challenges in childbirth and has the potential to cause perinatal morbidity and mortality [8, 12–14]. Evidence suggests that scaling up the management of diabetes in pregnancy can prevent stillbirths and deaths of mothers in South Africa [24], yet it does not receive much attention, and not much is known about its extent and distribution in South Africa. Research on diabetes in pregnant women in South Africa is limited and this hampers efforts to manage the disease [15].

South Africa is bound by legislation to commit towards realising the right to maternal health and the reduction of maternal mortality due to causes such as diabetes. Through provisions in its constitution, over the years the country has adopted a number of legislation and policies directed at reducing the maternal mortality rates such as the National Health Care Act, the Choice on Termination of Pregnancy Act and White Paper for the transformation of the health System which emphasised access to maternal, child and women’s healthcare services. Apart from these laws South Africa has adopted various Maternal Health Programmes such as the Campaign on Accelerated Reduction of Maternal, Newborn and Child Mortality (CARMMA) and Department of Health Strategic Plan for Maternal, Newborn, Child and Women’s Health (MNCWH) and Nutrition 2012–2016. Though it makes no mention of diabetes in pregnancy, the MNCWH emphasizes the need to tackle the social determinants of health in order to reduce maternal, newborn and child mortality [25]. In their guidelines for maternity care in South Africa the department of health reviews the treatment of diabetes during pregnancy and emphasises the need for tight control of blood glucose levels in pregnant women with pre-gestational and gestational diabetes [26]. The guidelines advise that all pregnant women with diabetes risk factors such as being older than 40 years, of Indian ethnicity, obese, those with a previous history of gestational diabetes and those with first degree relatives with diabetes should be screened. Although South Africa does not have any data on maternal mortality due to diabetes, notwithstanding this, the legislation, guidelines and clinical protocol provided by the department of health if applied stringently will help reduce maternal mortality. There are however weaknesses in clinical practice with regard to detection of diabetes in pregnancy, mainly as a result of late presentation. Treatment is also affected by late referral of patients [15]. This is a problem particularly in the public sector which is mainly used by a large section of the poor population. Future research is therefore imperative in order to influence policy refinement.

## Conclusion

This study contributes to the understanding of the extent of diabetes during pregnancy and its social determinants in South Africa. The identification of the risk factors associated with diabetes during pregnancy provides useful information that can be used in priority setting and planning for the improvement of maternal and child health. For instance the association between family history of diabetes and developing diabetes during pregnancy may imply that there is need to put in place measures that encourage healthy lifestyles for women with a family history of diabetes. Pre-pregnancy care for non-African women may help avert the adverse outcomes of diabetes during pregnancy.

Focusing on diabetes in pregnancy can help South Africa make that extra push required to reduce maternal and child mortality. South Africa did not meet its millennium development goals (MDGs) aimed at reducing maternal and child mortality by 2015, despite making much progress in the last decade [27]. As the country works towards meeting the new sustainable development goals set for 2030 [28], consideration should be made to managing non-communicable diseases such as diabetes, which can profoundly affect pregnancy outcomes [24].

## Abbreviations

CSDH: Commission on social determinants of health
EA: Enumeration area
GDM: Gestational diabetes mellitus
HSRC: Human sciences research council
MDG: Millennium development goals
SANHANES: South African National Health and Nutrition Examination Survey
SD: Standard deviation
VP: Visiting point
WHO: World Health Organization
